# The effects of obesity on thyroid function in a metabolically healthy high-fat, high-carbohydrate diet-induced obese rat model

**DOI:** 10.3389/fendo.2025.1538627

**Published:** 2025-04-22

**Authors:** Reveshni Pather, Andile Khathi, Phikelelani Ngubane

**Affiliations:** Department of Human Physiology, School of Laboratory Medicine and Medical Science, University of KwaZulu-Natal, Durban, South Africa

**Keywords:** obesity, thyroid, metabolically healthy obesity, high-fat high-carbohydrate diet, thyroid dysfunction

## Abstract

**Introduction:**

Obesity is a recognized exacerbator of thyroid dysfunction due to its detrimental effects on energy homeostasis, appetite regulation, basal metabolic rate, thermogenesis, and metabolism. However, almost all the reported findings on obesity-related thyroid dysfunction are based on models of metabolically unhealthy obesity (MUO) in the presence of insulin resistance. There are currently no reported studies using a metabolically healthy obesity (MHO) model characterized by the absence of insulin resistance to investigate thyroid dysfunction. Hence, this study aimed to investigate the association between thyroid dysfunction and obesity in a metabolically healthy high-fat high-carbohydrate diet-induced obese rat model.

**Materials and methods:**

Male Sprague Dawley rats were randomly divided into either the control diet or the high-fat high-carbohydrate diet group (HFHC) (n=9, per group). During the 5-month induction period, the control group did not develop obesity while consuming a standard diet with water. The HFHC diet group consumed the HFHC diet and water for the same duration and was diagnosed with obesity. Post-obesity confirmation, the animals continued with the respective diets for a further 7 months to maintain the obese state. Caloric intake, fasting blood glucose (FBG) and BMI were measured once a month for the duration of the experiment. Glucose homeostasis and thyroid functional parameters were assessed terminally, accompanied by satiety and pro-inflammatory markers.

**Results:**

The HFHC diet group presented with higher BMI, caloric intake and FBG, and elevated insulin, HOMA-IR, Hb1Ac, leptin and IL-6 levels compared to the control diet group. The HFHC diet group presented with significantly elevated levels of TSH, fT3 and fT4. These observations suggest that thyroid homeostasis is disturbed in the obese state. However, the reported elevated glycemic status indicators and IL-6 concentrations in the HFHC diet group did not satisfy the minimum criteria to be characterized as MUO.

**Conclusion:**

The HFHC diet has induced MHO in male Sprague Dawley rats. This warrants using this model to investigate the homeostatic changes that occur during the metabolically healthy obese state. This can open new avenues for developing preventative measures to avoid progressing to MUO.

## 1 Introduction

The obesity epidemic is one of the most frequent pathologies affecting the general population in the 21^st^ century (1). In 2022, the World Health Organization (WHO) reported an unprecedented increase in the prevalence of obesity globally, particularly in middle and low-income countries (2, 3). Globally, the World Obesity Atlas (2022) estimated that by 2030, one billion people will be living with obesity (2). The enhanced prevalence is due to a combination of low physical activity and dietary choices associated with modern sedentary lifestyles, such as the chronic consumption of high-caloric diets rich in carbohydrates and saturated and polyunsaturated fats (4–6). Consequently, the excessive dietary intake of calorie-dense and nutrient-poor foods has led to the inefficient metabolism of carbohydrates and fats (7, 8). Hence, this results in the ectopic accumulation of triglycerides and free fatty acids in non-adipose depots, contributing to chronic cellular dysfunction and injury (9, 10). Obesity is associated with chronic comorbidities such as diabetes mellitus, cardiovascular disease, kidney disease and dyslipidemia (11, 12). The risk for these non-communicable diseases rises as body mass index (BMI) increases (2, 3). Moreover, central obesity is linked to endocrine abnormalities, including thyroid dysfunction, either by a direct increase in organ fat content or indirect metabolic changes (13, 14).

The thyroid is a vital multifunctional endocrine gland that mainly facilitates the regulation of metabolism (15, 16). This butterfly-shaped gland is responsible for the production and release of two thyroid hormones, Thyroxine (T_4_) and Triiodothyronine (T_3_) (17). These hormones are produced and stored in the follicular epithelial cells of the thyroid gland (17). Thyroid hormone secretion is regulated by the hypothalamus and the pituitary gland in the brain (18, 19). The hypothalamus secretes thyroid-releasing hormone (TRH), which stimulates the pituitary gland to secrete thyroid-stimulating hormone (TSH) (17). TSH stimulates the thyroid follicular cells to release T_4_ and T_3_ (17, 20). T_3_ and T_4_ signaling modulate energy expenditure at various levels through peripheral and central mechanisms (15). Additionally, thyroid hormones regulate the function of multiple organs and tissues, including the liver, heart, pancreas, skeletal muscle and adipose tissue (15, 21).

Over the years, numerous studies have reported the complexity of the relationship between obesity and the hypothalamic-pituitary-thyroid axis. In literature, thyroid dysfunction has been widely reported to be associated with obesity as it has multiple effects on energy homeostasis, appetite, free fatty acid oxidation, basal metabolic rate, thermogenesis and metabolism (20–22). Alterations of these hormones have been demonstrated to correlate with increased body mass index (BMI), insulin resistance and hypertension, which are indispensable features comprising obesity (22, 23). BMI and increased waist circumference have been negatively associated with serum-free T_4_ (fT_4_), and fat accumulation has been associated with lower fT_4_ and higher TSH levels among overweight and obese individuals, thereby resulting in a positive correlation between TSH and the progressive increase in body weight over time (18, 20, 23). Adipocytes are an active endocrine organ as they produce leptin, a satiety hormone (24, 25). Circulating leptin levels reflect adipose tissue reserves and correlate with the degree of obesity (20, 26). Leptin is also a neuroendocrine regulator of the hypothalamic-pituitary-thyroid axis by directly regulating TRH gene expression and, subsequently, TSH and thyroid hormone levels (18). Insulin resistance, a hallmark of obesity, can impair the conversion of inactive T_4_ to active T_3_ in peripheral tissues, leading to decreased cellular T_3_ availability (27, 28). The hormonal conversion is important for optimal thyroid hormone activity and overall metabolism; hence, impaired T_3_ production can reduce metabolic rate, leading to weight gain, dyslipidemia and ectopic fat deposition (27, 29). Furthermore, reduced thyroid hormone conversion leads to further dysregulation of insulin secretion, thus exacerbating tissue insulin insensitivity (30).

Majority of the reported findings on obesity-related thyroid dysfunction are based on models of metabolically unhealthy obesity (MUO) in the presence of insulin resistance. MUO refers to individuals who are obese with insulin resistance and exhibit one or more metabolic abnormalities associated with obesity, such as dyslipidemia, inflammation, hypertension, and glucose intolerance (31, 32). The findings of these studies are beneficial to a large majority of the obese population, but these particular studies fail to include the metabolically healthy obese subtype. The metabolically healthy obesity (MHO) subtype comprises approximately 30% of the total obese population and is characterized by preserved insulin sensitivity and lowered risk for cardiometabolic complications (33, 34). Even though a significant portion of the global obese population falls under the MHO subtype, there is a paucity of reported studies using a diet-induced MHO model to investigate thyroid dysfunction. Hence, the mechanisms through which thyroid hormone levels are dysregulated with prolonged high-caloric feeding have not been fully established in an insulin-sensitive diet-induced obese model.

Current animal models have delivered invaluable information to our knowledge of the pathophysiology of obesity (35–37). Although genetically modified obese animal models reproduce various metabolic abnormalities occurring in overweight/obese patients, the development of diet-induced obese animals has aided in identifying the pathobiology and metabolic features associated with different stages of obesity-related disease progression in humans (36–38). Furthermore, researchers have observed distinct patterns of voluntary food consumption in genetically modified obese animals versus diet-induced animal models (12, 39). Various studies have concluded that this might influence the accuracy of the experimental results obtained from genetically modified models, as most genetic models fail to emulate the critical metabolic characteristics of human obesity and the natural patterns of disease initiation and development in humans (12, 37, 38). Diet-induced models such as the high-carbohydrate diet, the high-fructose/sucrose diet, the high-fat diet and the combination of high-fat high carbohydrate supplemented with fructose diet have all been previously used to create glucose intolerant/insulin-resistant obese animal models with altered thyroid function (40–44). Studies conducted using a high-fat lard diet increased triglyceride levels in serum, and decreased serum TT_4_ and fT_4_ levels in parallel with elevated serum TSH levels (44, 45). A high-fructose diet-induced animal study demonstrated that fructose intake altered thyroid homeostasis by modifying the expression of genes related to lipogenesis in the thyroid gland and genes associated with thyroid hormone metabolism (43). Furthermore, reduced serum TT_3_ and fT_3_ levels were also reported in high-fructose fed rats (43). Similarly, high-sucrose diet studies noted significant decreases in serum T_3_, fT_3_ and T_4_ (46). Short-term studies using a high-fat simple carbohydrate diet demonstrated increases in T_3_ and TSH, while TRH gene expression was decreased (47). The diet-based models mentioned above have successfully induced obesity in isolation, but these diet models do not reflect modern dietary patterns.

Hence, to date, there is still a paucity of reported studies on thyroid dysfunction using an obese model that genuinely mimics the combined high-fat and high-carbohydrate content popular in modern dietary patterns. Therefore, there is still a need to characterize and outline hypothalamic-thyroid function in a diet-induced metabolically healthy obese model. In an effort to fill this gap in the literature, this study aimed to investigate the effects of metabolically healthy obesity on thyroid function markers and to determine the association between thyroid dysfunction and obesity in a metabolically healthy high-fat high-carbohydrate diet-induced obese rat model.

## 2 Materials and methods

### 2.1 Chemicals and reagents

Phosphate buffered saline (PBS), hydrochloric acid (HCl) (Merck, Wadeville, South Africa), and Isofor inhalation anesthetic (Safeline Pharmaceuticals (Pty) Ltd., Roodepoort, South Africa). All other chemicals and reagents were of analytical grade and sourced from standard commercial suppliers.

### 2.2 Animals and housing conditions

18 male Sprague Dawley rats weighing 180-200g were used in this study. They were bred and housed in Makrolon polycarbonate metabolic cages (Techniplast, Labotec, South Africa) in the Biomedical Resource Unit (BRU) breeding colony at the University of KwaZulu-Natal. The rats were kept under standard laboratory conditions, which included constant temperature (22 ± 2°C), CO_2_ content (<500 p.p.m), relative humidity of 55 ± 5%, noise levels less than 65 decibels and illumination (12-hour light/dark cycle (lights on at 7h00)). The animals were allowed access to food and water *ad libitum*. Fine wood shavings were used for bedding. Environmental enrichment included various dimensions of PVC pipe (Techniplast, Labotec, South Africa). All materials, including bedding, PVC pipes, lids and bottles were autoclaved before use. Prior to the commencement of the experiment, the rats were acclimatized to their new environment for one week while consuming standard rat chow (Meadow Feeds, Pietermaritzburg, South Africa) and water before exposure to the experimental high-fat high carbohydrate (HFHC) diet (AVI Products (Pty) Ltd., Waterfall, South Africa) to induce obesity. The sample size calculation was conducted to obtain the desired sample size necessary to induce obesity and its complications in a male Sprague Dawley rat model. A single-blinded technique was followed to randomly divide the animals into experimental groups to prevent bias. The animals were divided into separate groups by the animal technician, who was not involved in the study. The animals were kept in their specific group cages throughout the experimental period and never mixed.

### 2.3 Ethics statement

This study was reviewed and approved by the Animal Research Ethics Committee (AREC) of the University of KwaZulu-Natal, Durban, South Africa (AREC/026/020M). All animal housing arrangements and experimental procedures were according to the ethics and animal care guidelines set out by the Animal Research Ethics Committee (AREC) of UKZN, Durban, South Africa. Animals were constantly monitored for pain and discomfort according to the criteria set out by the Animal Research Ethics Committee’s humane endpoint document, which complies with the ARRIVE guidelines.

### 2.4 Diet composition

A high-fat, high-carbohydrate diet was chosen because it resembles modern dietary choices (48). The detailed composition of the customized experimental high-fat high-carbohydrate (HFHC) and the control diet can be seen in **Table 1** and **Table 2**. Differences in dietary composition between the control diet and the high-fat high-carbohydrate diet can be seen in **Table 3**.

**Table 1:** Composition of the high-fat high carbohydrate diet (HFHC).

Ingredient	Incl (%)	Mix(kg)
Maize	38.98	390.000
Palm Oil	20.99	210.000
Soya Full Fat	14.99	150.000
Wheat Gluten	6.50	65.000
Flour	6.00	60.000
Monodex	5.00	50.000
Sugar – Sucrose	5.00	50.000
Limestone	1.00	10.000
Dicalcium phosphate	0.50	5.000
Vitamin Premix	0.35	3.500
Salt- Fine	0.30	3.000
Amino Acid- DL-Methionine	0.30	3.000
Mineral Premix	0.10	1.000
	**100.01**	**1000.50**

**Table 2:** Composition of the control rat diet.

Main ingredients	Min/Max	Mix (g/kg)
Protein	Min	180
Moisture	Max	120
Fat	Min	25
Fibre	Max	60
Calcium	Max	18
Phosphorus	Min	7
	Total	410
Manufacturer (Epol) ingredient statement:		

This animal feed contains: Grain and grain by-products, roughages, plaint protein products, oils and fats, sugar cane by-products, amino acids, minerals, vitamins, enzymes and pellet binders. This product contains genetically modified ingredients.

**Table 3:** Differences in the composition of carbohydrates, fats, and proteins in the control and high-fat, high-carbohydrate (HFHC) diet.

	Control diet (% Kcal/g)	HFHC diet (% Kcal/g)
Carbohydrates	55	55
Fats	16	30
Proteins	29	15

### 2.5 Experimental design

18 male Sprague Dawley rats were randomly divided into two groups, namely the control group (n=9) and the high-fat, high-carbohydrate (HFHC) diet (n=9) group. The control group consumed a standard rat chow diet and water for 5 months and did not become obese, while the HFHC group consumed the customized experimental diet and water for the same duration and was diagnosed with obesity. The high-fat, high-carbohydrate group was diagnosed with obesity following positive results from animal BMI above 1.3 g/cm^2^ (4, 12). Both groups continued with their respective diets for a further 7 months to maintain obese status. The experimental period was 12 months, which included the diet adaptation periods.

Body weights were measured using a portable digital scale. To determine food and water intake (caloric intake), the animals were placed overnight in individual metabolic cages (Techniplast, Labotec, Cape Town, South Africa). Fasting blood glucose (FBG) concentrations were measured using a OneTouch select glucometer (Lifescan, Mosta, Malta, United Kingdom). Caloric intake, FBG concentrations, body weights and lengths were measured once a month for the duration of the experiment. Baseline measurements for both diet groups can be found in **Supplementary Table 1**.

### 2.6 Experimental procedures

#### 2.6.1 Body mass index

BMI (g/cm^2^) was calculated once a month for the duration of the experimental period. A BMI between 0.45 – 0.68 g/cm^2^ was considered normal, 0.69-1.2 g/cm^2^ was overweight, and a BMI above 1.3 g/cm^2^ was obese (4, 12). The BMI was calculated using the following formula:


$$Body\;mass\;index=\left(\frac{Weight\;\left(g\right)}{Lenght\;\left(cm^{2}\right)}\right)$$

#### 2.6.2 Calculation of food and water intake

The food and water intake levels were recorded once a month for the duration of the experimental period. The animals were placed in specialized metabolic cages to measure food and water intake for 24 hours. Each animal was given 100 g of their respective diet and 100 mL of water. After 24 hours, the remaining grams of food and the volume of water that the animals had not consumed were measured to determine caloric intake.

#### 2.6.3 Fasting blood glucose concentration

FBG concentrations were measured in all animals once a month for the duration of the study using a previously well-established laboratory technique (49). Briefly, all animals were fasted for 18 hours, and at the end of the fasting period, blood was drawn using the tail prick method. The fasting blood glucose concentrations were measured using a OneTouch select glucometer (Lifescan, Mosta, Malta, United Kingdom).

### 2.7 Terminal studies

At the end of the experimental period, all animals were anaesthetized by exposure to Isofor (100 mg/kg) (Safeline Pharmaceuticals (Pty) Ltd, Roodepoort, South Africa) for 3 minutes via an anesthetic gas chamber (Biomedical Resource Unit, UKZN, Durban, South Africa). The rats were anaesthetized prior to the performing a cardiac puncture due the invasive nature of the blood collection technique. While the rats remained unconscious, blood was collected via cardiac puncture and injected into pre-cooled EDTA tubes. Exsanguination due to cardiac puncture was the cause of death and was confirmed by the veterinarian. The collected blood samples were centrifuged (Eppendorf centrifuge 5403, Hamburg, Germany) at 4°C, 503 g for 15 minutes. Plasma was collected and stored at -80°C in a Bio Ultra freezer (Snijders Scientific, Tilburg, Holland) until it was required for biochemical analysis. Plasma samples were used to measure thyroid stimulating hormone, free Triiodothyronine, free Thyroxine, insulin, interleukin-6, glycated hemoglobin, and leptin concentrations.

### 2.8 Biochemical analysis

Glycated hemoglobin (HbA1c) (Rat glycated hemoglobin A1c (HbA1c); Lot no: L240904063; Manufacturer: Cloud-Clone Corp) was terminally analyzed in plasma according to the manufacturer’s instructions using a rat-specific Competitive ELISA kit (Cloud-Clone Corp, Katy, Texas, USA). Plasma free Triiodothyronine (fT_3_) (fT3(free Triiodothyronine) ELISA KIT; Catalog no: E-EL-0079; Manufacturer: Elabscience) and free Thyroxine (fT_4_) (fT4 (free Thyroxine) ELISA KIT; Catalog no: E-EL-0122; Manufacturer: Elabscience) were terminally analyzed using separate rat-specific Competitive ELISA kits according to the manufacturer’s instructions (Elabscience Biotechnology Co., Ltd., Wuhan, China). Thyroid stimulating hormone (TSH) (Rat TSH(Thyroid stimulating hormone) ELISA KIT; Catalog no: E-EL-R0976; Manufacturer: Elabscience), leptin (Leptin ELISA KIT; Catalog no: E-EL-R0582; Manufacturer: Elabscience), insulin (Rat INS(Insulin) ELISA KIT; Catalog no: E-EL-R3034; Manufacturer: Elabscience) and interleukin-6 (Rat IL-6(Interleukin-6) ELISA KIT; Catalog no: E-EL-R0015; Manufacturer: Elabscience) were terminally analyzed in plasma using separate rat-specific Sandwich ELISA kits according to the manufacturer’s instructions (Elabscience Biotechnology Co., Ltd., Wuhan, China). The protocols for TSH, insulin, IL-6 and leptin were the same as they all required sandwich ELISA kits. Glycated hemoglobin and thyroid hormones required competitive ELISA kits, and the procedure was still the same except for the incubation periods, as the competitive elisa required less time for incubation. Briefly, the kits included a micro-ELISA plate that was coated with antibodies specific to each of the parameters measured.100 µL of standards or samples were added to the pre-coated wells and incubated for 90 minutes at 37°c. Post 90 minutes, the liquid was discarded and 100 µL of biotinylated detection antibody (Ab) was added to each well using a multi-channel pipette and incubated for 60 minutes at 37°c. After incubation, the liquid was aspirated and subjected to 3 times washes using a wash buffer. 100 µL of avidin-horse radish peroxidase (HRP) conjugate was added and allowed to incubate for 30 minutes at 37°c. The microplate was subjected to 5 x washes, and 90 µL of substrate reagent was added, followed by a 15-minute incubation period at 37°c. 50 µL of stop solution was then added, and the optical density was measured at 450 nm using the Spectrostar Nano spectrophotometer (BMG Labtech, Ortenburg, Germany). The standard curve was determined using blank corrected data of the known standard concentrations for the sandwich elisa. The competitive elisa data was not blank corrected because the assay relies on the relative signal (or lack thereof) from the enzyme-labelled antigen, which is directly proportional to the amount of analyte (antigen) in the sample rather than the absolute signal. The sample concentrations of glycated hemoglobin, plasma TSH, fT_3_, fT_4_, leptin, IL-6, and insulin were extrapolated from their respective standard curves.

### 2.9 HOMA2-IR index

The homeostatic model assessment (HOMA) is an authenticated technique that measures β-cell function and insulin resistance from fasting glucose and insulin concentrations (50). The relationship between glucose and insulin in the basal state reflects the balance between hepatic glucose output and insulin secretion, which is maintained by a feedback loop between the liver and the β-cells. The HOMA2-IR index was the model of choice because it was updated with some physiological adjustments to a computer version, yielding a more accurate index relative to the initial HOMA1-IR that was published by Levy et al. in 1998 (51). A range of 0.5-1.4 indicates a standard HOMA-IR value. An index value less than 1.0 means the insulin sensitivity is optimum. On the other hand, index values above 1.9 indicate early insulin resistance, and those above 2.9 indicate significant insulin resistance. Therefore, this study utilized the HOMA2-IR index to assess the insulin sensitivity of male Sprague Dawley rats. HOMA2-IR was calculated according to the formula:


$$HOMA-IR=\;\frac{\text{Fasting plasma insulin }\left(\frac{\text{μU}}{\text{ml}}\right)\text{ x Fasting plasma glucose }\left(\frac{\text{mmol}}{\text{L}}\right)}{\left(22.5\right)\;}$$

### 2.10 Statistical analysis

All data were expressed as means ± standard error of the mean (SEM). Normality tests were performed, and GraphPad Prism Instant Software for Windows (version 5, GraphPad Software; San Diego, California, USA) was used for statistical comparisons. The student t-test was used to analyze the statistical differences between the control and high-fat high-carbohydrate diet group for FBG concentrations and terminal parameters. BMI and caloric intake was analyzed via a one-way Analysis of Variance (ANOVA) test followed by Bonferroni’s multiple comparison *post hoc* test. A value of p< 0.05 was considered statistically significant.

## 3 Results

### 3.1 Body mass index and body weight

**Figure 1:**
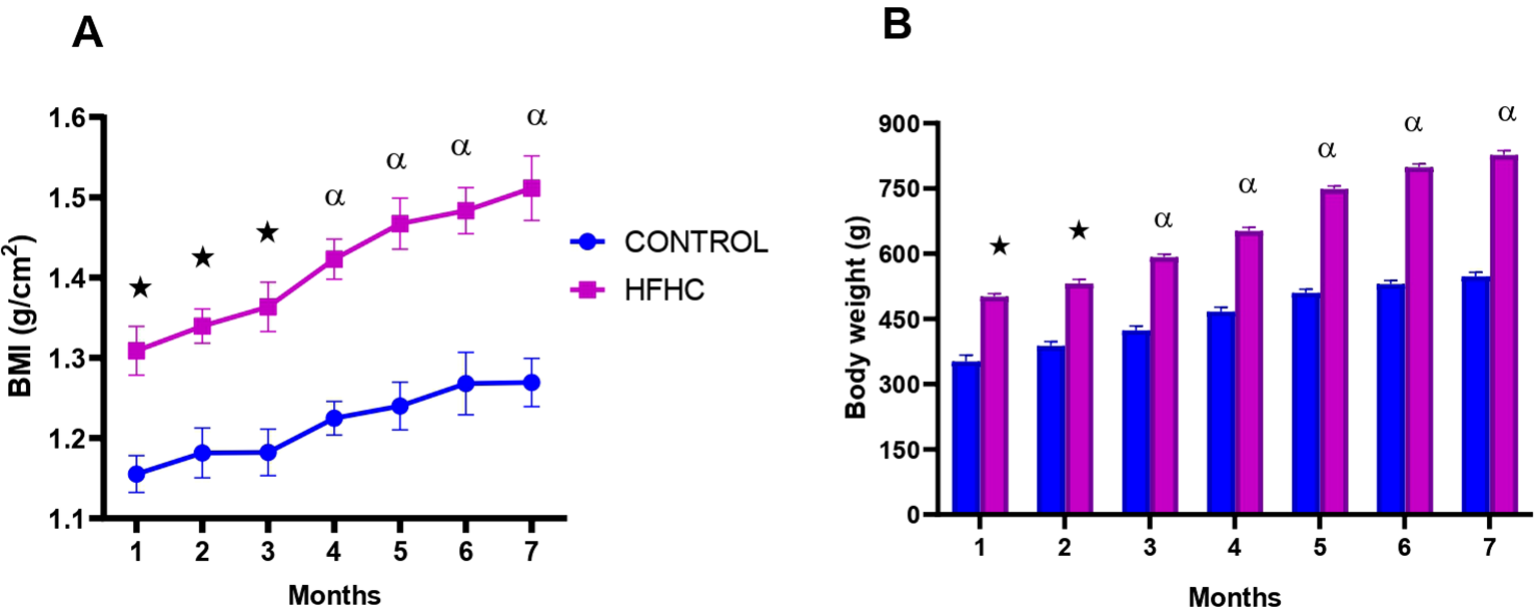
The monthly body mass index **(A)** and body weight **(B)** calculated in the control and high-fat, high-carbohydrate (HFHC) diet groups for 7 months post obesity induction. Values are expressed as mean ± SEM (n=9 in each group). ★ Denotes p<0.05, and α denotes p<0.01 when compared to the control diet group.

**Figure 1** depicts the monthly body mass index (**Figure 1A**) and body weight (**Figure 1B**) in the control diet group (n=9) and high-fat, high carbohydrate (HFHC) diet group (n=9) for 7 months, post obesity induction. The HFHC diet group presented with significantly higher monthly BMI and body weight when compared to the control group post-obesity induction **★** (control vs HFHC, p<0.05, **Figure 1**) and α (control vs HFHC, p<0.01, **Figure 1**).

### 3.2 Caloric intake

**Figure 2:**
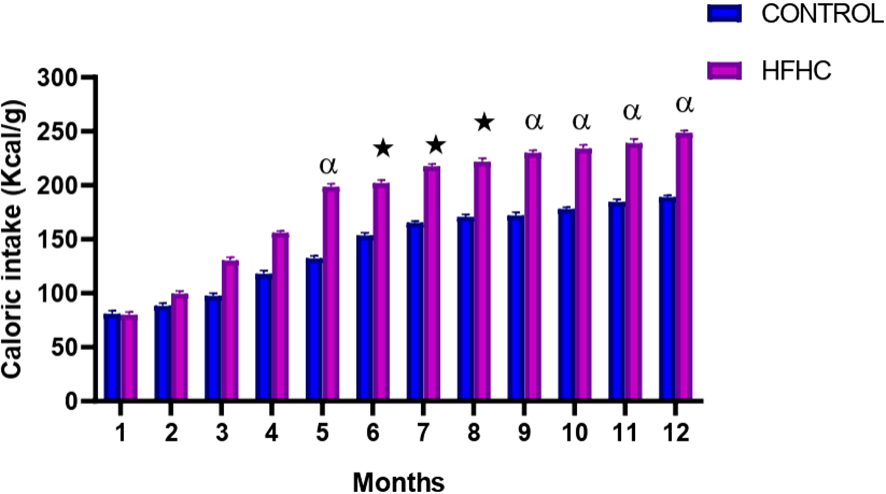
The monthly caloric intake calculated in the control and high-fat high-carbohydrate (HFHC) diet groups form induction to termination. Values are expressed as mean ± SEM (n=9 in each group). ★ Denotes p<0.05, and α denotes p<0.01 when compared to the control diet group.

Food consumption was monitored monthly during the experimental period which lasted 12 months. The results showed that from month 5 until termination, the HFHC diet group had a significantly higher caloric intake in comparison to the control diet group **★** (control vs HFHC, p<0.05, **Figure 2**) and α (control vs HFHC, p<0.01, **Figure 2**). No significant differences were observed between the diets groups between months 1 to 4 of the induction phase.

### 3.3 Glycemic status

Glycemic status indicators (fasting blood glucose (FBG) concentrations, insulin concentrations, HOMA-IR and glycated hemoglobin) were measured in the control diet group (n=9) and the high-fat, high-carbohydrate (HFHC) diet group (n=9) after the experimental period. It was evident (**Table 4**) that the plasma concentrations of fasting blood glucose (p<0.0001) and insulin (p<0.01) were significantly higher in the HFHC diet group than in the control diet group. This was accompanied by elevated levels of glycated hemoglobin (p<0.01) in the HFHC group. Furthermore, the HFHC group had significantly higher HOMA-IR value (p<0.0001) compared to the control group, which was above the optimum sensitivity range (1.0) but below the early insulin resistance range (>1.9). Whereas, the control group HOMA-IR value was within the insulin sensitive range (1.0).

**Table 4:** The effects of diet-induced obesity on glycaemic status indicators.

Diet group (n=9)	Control	HFHC	P-value
Plasma glucose (mmol/L)	4.40 ± 0.12	5.12 ± 0.20 •	<0.0001
Plasma insulin (μU/ml)	5.2 ± 0.41	7.1 ± 0.16 α	<0.01
HOMA2-IR values	1.0 ± 0.05	1.6 ± 0.07 •	<0.0001
Hb1Ac (µg/ml)	167.66 ± 0.18	209.74 ± 0.29 α	<0.01

Values are expressed as mean ± SEM (n=9 in each group). • Denotes p<0.0001 and **α** denotes p<0.01 when compared to the control diet group.

### 3.4 Thyroid functional indicators

Thyroid stimulating hormone (TSH), free Triiodothyronine (fT_3_), free Thyroxine (fT_4_) and fT_3_/fT_4_ ratio were terminally measured in the control diet group (n=9) and the high-fat, high-carbohydrate (HFHC) diet group. It was evident (**Table 5**) that the plasma concentrations of thyroid stimulating hormone (p<0.01), free Thyroxine (p<0.0001), free Triiodothyronine (p<0.05) and fT_3_/fT_4_ ratio were significantly higher in the HFHC diet group than in the control diet group.

**Table 5:** Terminal concentrations of thyroid functional status indicators.

Diet group (n=9)	Control	HFHC	P-value
TSH (ng/ml)	15.4 ± 0.33	25.4 ± 0.01 α	<0.01
fT_3_ (pmol/L)	3.66 ± 0.73	6.25 ± 1.11 ★	<0.05
fT_4_ (pmol/L)	55.95 ± 3.83	62.63 ± 5.74 •	<0.0001
fT_3_/fT_4_ ratio	0.066 ± 0.41	0.09 ± 0.97 ★	<0.05

Values are expressed as mean ± SEM (n=9 in each group). • Denotes p<0.0001, **α** denotes p<0.01 and ★ denotes p<0.05 when compared to the control diet group.

### 3.5 Interleukin six and leptin

**Figure 3:**
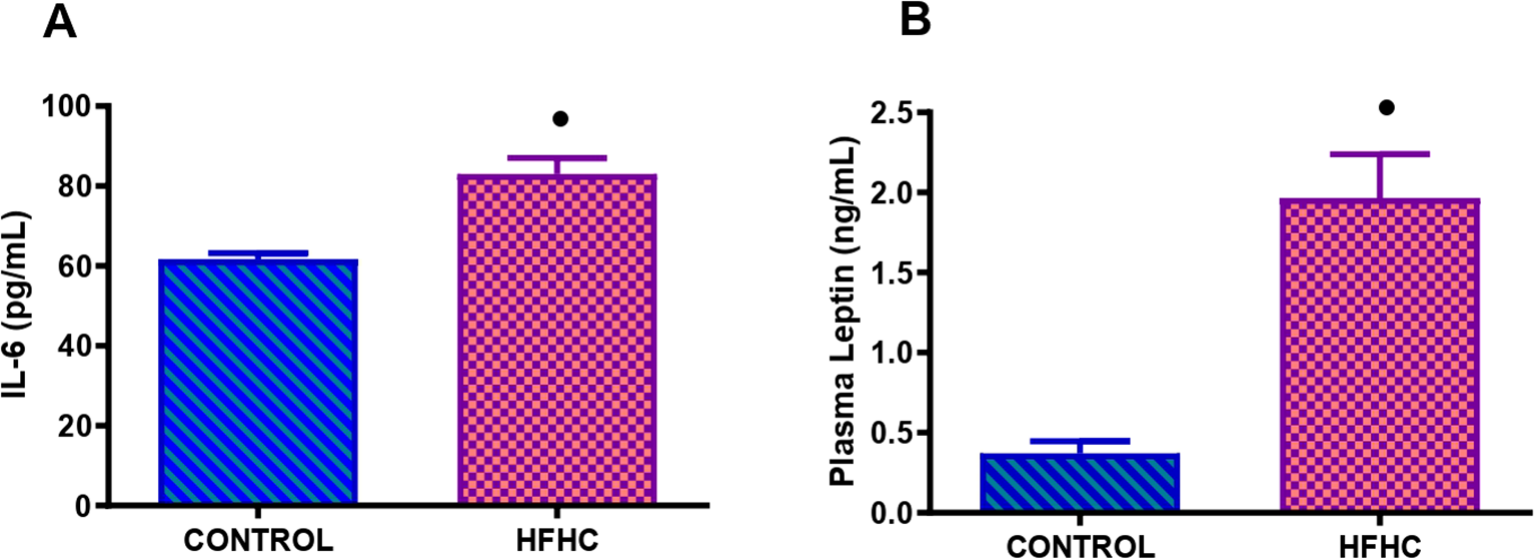
Terminal concentration of IL-6 **(A)** and leptin **(B)** in the control and high-fat, high carbohydrate (HFHC) diet group. Values are expressed as mean ± SEM (n=9 in each group). **•** Denotes p<0.0001 when compared to the control diet group.

**Figure 3** displays the plasma IL-6 (**Figure 3A**) and leptin (**Figure 3B**) concentrations measured in the control diet group (n=9) and the high-fat, high-carbohydrate (HFHC) diet group (n=9) after the experimental period. The HFHC diet group concentration of plasma IL-6 and leptin was significantly higher than the control diet group **•** (control vs HFHC, p<0.0001, **Figure 3**).

## 4 Discussion

This study investigated the possible alterations to thyroid function markers and determined the association between thyroid dysfunction and obesity in the absence of insulin resistance in a high-fat high-carbohydrate diet-induced metabolically healthy obese rat model. The present study aimed to develop an animal model of metabolically healthy obesity (MHO) that would imitate the natural history and the metabolic characteristics of the human syndrome. This study aimed to develop an animal model that is neither inherited nor genetically insulin resistant and is easily accessible, economical, and with a high success rate. The results in this study demonstrated that this model displays obesity-related characteristics, which include weight gain, hyperphagia, slight insulin insensitivity and altered inflammatory profile over time (52–54). Also, in the literature, obesity is characterized by moderate hyperglycemia, hyperphagia, hyperlipidemia and altered metabolic profile (55, 56).

Obesity is described as a combination of excessive fat accumulation in adipose tissue and insulin resistance; thus, it is associated with unhealthy eating habits and weight gain (13, 57). Calorie intake regulation is crucial in maintaining a healthy weight and insulin sensitivity (58). In this study, the prolonged exposure of the animals to a high-fat, high-carbohydrate diet resulted in increased food intake and caloric intake, which resulted in a higher body weight gain in the HFHC group compared to the control group. Furthermore, an increase in BMI is expected during obesity because insulin insensitivity is still at a moderate level in the preliminary stages of chronic high-caloric food consumption. Therefore, insulin is still effective due to the compensatory mechanism of the pancreatic β-cells that release more insulin into the blood, and insulin as an anabolic hormone endorses glucose storage as glycogen in the liver and fat storage in adipose tissue (59, 60). The outcome of this is inevitably weight gain, as demonstrated by increased BMI and body weight post-obesity confirmation. It was also noted that the BMI and body weight values of the control diet group continued to increase at the latter end of the study, which was unusual. However, after analysis of the body weight and caloric intake results, it can be deduced that the slight increase in weight gain and caloric intake may be due to a combination of reduced metabolism and reduction in growth rate as the rats are senior adults. This can be validated by the plateau formed in the control group’s BMI graph. This indicates that the rats are presumably growing in circumference but not in length. Additionally, the significant increase in caloric intake, BMI, and body weight post-obesity induction in the HFHC group indicates that this dietary model was successful in inducing obesity and maintaining the obese status until termination.

Metabolically unhealthy obesity (MUO) is characteristically associated with adipocyte hypertrophy, abnormal lipid profile and impaired glucose tolerance (32, 61). Most notably, the primary catalyst in the development of MUO is the prolonged consumption of calorie dense diets enriched with poor glycemic indexed components (28, 62). Literature has reported that chronic consumption of high-caloric diets increases blood glucose levels beyond the threshold for normal glucose homeostasis, consequently leading to a dysfunctional glucose homeostatic state and insulin insensitivity due to overburdened energy storage depots (63–65). In the current study, we reported on fasting blood glucose, insulin, glycated hemoglobin concentrations and HOMA2-IR index, as seen in **Table 4**, which were notably higher in the HFHC diet group than in the control group. These results are consistent with previous diet-based studies, which also reported increases in plasma glucose, HOMA-IR index and Hb1Ac concentrations alongside increased caloric intake and weight gain with the chronic consumption of high-caloric diets (41, 42, 44, 66). However, in contrast to previous studies, the plasma insulin concentration in the present study was significantly higher in the HFHC group than the control group but not at the threshold to cause alterations to thyroid hormone levels, as demonstrated in models where insulin resistance is present (67, 68). Furthermore, the Hb1Ac results in this study, align with past diet-based research conducted in our laboratory that reported that elevated plasma glucose concentrations result in non-enzymatic glycation of hemoglobin (66, 69). Throughout a red blood cell 120-day lifecycle, a persistent increase in blood glucose concentration, as in the case of insulin resistance, has been demonstrated to cause glucose-mediated non-enzymatic glycation of hemoglobin via the Amadori reaction, forming a permanently irreversible ketoamine linkage (66). As HbA1c production is directly proportional to the concentration of circulating blood glucose and survives for the lifespan of the red blood cell, it is regarded as an excellent index for evaluating long-term glycemic control (70). However, even though the HFHC diet group results were significantly higher than the control group, the concentrations displayed were below the minimum criteria for diagnosing glucose intolerance as stated by the American diabetes association (71). This includes normal regulation of glucose homeostasis indicated by fasting blood glucose (FBG) below 5.6 mmol/L, postprandial glucose level of less than 7.8 mmol/L and glycated hemoglobin below 5.7% (72–74). The HOMA-IR index of the HFHC diet group was within the insulin sensitivity range (<1.9) even though it was higher than the control group. This was accompanied by an Hb1Ac concentration of less than 5.7%. Interestingly, this study’s HOMA-IR index and Hb1Ac results characterize the metabolically healthy obese state. The metabolically healthy obese subtype is characterized by preserved insulin sensitivity and lowered risk for cardiometabolic complications (33, 34). Overall, the glycemic status markers investigated in this study indicate that the HFHC diet group is still insulin-sensitive even after chronic exposure to the high-energy diet. From the literature evidence, it is known that diet-induced insulin resistance exacerbates thyroid dysfunction (44, 75). Hence, this could be the first indication of how the metabolically healthy obese state may aid in delaying the onset of thyroid homeostatic dysregulation and overt MUO related comorbidities.

Obesity and thyroid hormones are interconnected in several ways, and changes in their bidirectional relationship can significantly impact body weight and metabolism (15, 18). The results of the current study show that the chronic ingestion of a HFHC diet led to a gradual increase in BMI and body weight in the HFHC group, post obesity conformation, compared to the control group. This is indicative of an altered homeostatic state. BMI and increased waist circumference have been negatively associated with serum-free T_4_ (fT_4_), and fat accumulation has been associated with lower fT_4_ and higher TSH levels among overweight and obese individuals, thereby resulting in a positive correlation between TSH and the progressive increase in body weight over time (18, 20, 23, 30). In this study, the plasma TSH and fT_3_ concentrations were significantly higher in the HFHC group than in the control group. These results corroborate previous findings showing elevated plasma TSH, fT_3_ and BMI in obese patients compared to normal healthy weighted individuals (23, 76, 77). Various obesity-based human studies have attributed the increase in TSH levels to either increased leptin production by adipose tissue or as an adaptive response to increase energy expenditure (78–80). Obesity-induced hyperleptinemia can stimulate the pituitary gland to increase TSH release and subsequently free thyroid hormone secretion (22, 81, 82). This could be interpreted as the body’s defense mechanism to counteract weight gain due to hyperphagia (78, 79). This study’s findings agree with previous literature as the significantly elevated leptin, TSH, and fT_3_ concentrations in the HFHC group mirror the results of human and animal-based obesity studies (22, 47, 78). MUO studies using human and animal models have reported low-normal fT4 with elevated TSH and fT3 concentrations in the obese groups (44, 47, 78, 79, 83). In contrast, the elevated fT4 concentration in the HFHC group in the present study was unusual, as almost all obesity-related studies have reported a negative correlation between increasing BMI and fT4 levels (23, 76, 77). Suppose this anomaly is looked at in terms of MUO studies. In that case, the statistically significant but comparatively small elevation in fT4 concentration in the HFHC group in this study may provide a clue on the counteracting mechanisms involved in the metabolically healthy obese state. In MUO studies, low fT4 levels reported in the obese group are almost always associated with higher glucose levels and insulin resistance (30, 44, 45). The combination of obesity and insulin resistance-induced TSH secretion stimulates T3 production from T4 (84). The net result is low fT4 and high fT3 with relatively high TSH levels (84, 85). Furthermore, a human-based study of various obesity phenotypes has noted a decrease in thyroid hormone receptors in the obese state; hence, both TSH and peripheral fT3 concentrations increase, reducing the negative feedback mechanism (86). The MHO model used in this study presented preserved insulin sensitivity, which was evidenced by the HOMA-IR index values within the insulin sensitivity range (**Table 4**). Literature has indicated that fT4 levels are negatively associated with the HOMA-IR index (83, 87). Additionally, an obesity study by Amouzegar et al., 2015 using different obesity phenotypes reported that the MHO group had increased TSH and thyroid hormone levels compared to metabolically obese individuals (83). These findings agree with the present study, as the concentration of TSH and fT4 were significantly elevated in the HFHC diet group compared to the control diet group. Elevated fT4 levels reported in this study could also be due to alterations in the conversion of T4 to the active T3 form in the body (76, 88). Increased fT4 production could be speculated to be a consequence of various hormonal and compensatory factors driving the obesity conundrum. FT_3_/fT_4_ ratio, which is used to evaluate the conversion rate of fT_4_ to fT_3_ and as a measure of thyroid hormone activity, was significantly elevated in the HFHC group. Obesity induced increases in fT_3_/fT_4_ ratio is possibly due to intensified deiodinase activity as a compensatory mechanism against fat accumulation (80). Furthermore, a higher fT_3_/fT_4_ ratio indicates greater peripheral thyroid hormone sensitivity and is linked to decreased cardiometabolic risks and mortality (89, 90). This is interesting as cardiometabolic risk factors are the fundamental components that differentiate MUO and MHO (31, 32, 34). The elevated fT_3_/fT_4_ ratio in the HFHC diet group could explain how the metabolically healthy obese state aids in maintaining homeostasis to an extent. Further investigation is required as it could assist in improving the early diagnosis of diseases in the metabolically obese state.

Chronic low-grade inflammation associated with obesity can interfere with the functioning of the thyroid gland and the conversion of thyroid hormones (22, 82). Inflammatory cytokines can affect the growth and differentiation of thyroid follicular cells, leading to the abnormal synthesis and secretion of thyroid hormones (22, 82). Interleukin-6 (IL-6) is considered an inflammatory and anti-inflammatory adipokine since macrophages and T-cells release it in adipose tissue from obese individuals (91). Its release occurs in a size-dependent manner (i.e., larger adipocytes release higher amounts of IL-6) and links obesity to a state of low-grade inflammation (91, 92). This was observed in the HFHC diet group, as there was a significant increase in IL-6 expression compared to the control group. This was further evidenced by the significant increase in body weight, BMI and calorie intake in the HFHC diet group, which indicates increased adipose tissue size and weight observed in obese individuals. Studies suggest that IL-6 potentiates the action of leptin as IL-6 receptors are also expressed in several areas of the brain, such as the hypothalamus, in which it controls appetite and energy regulation (92–94).

Hormone leptin, is a neuroendocrine regulator of the hypothalamic-pituitary-thyroid (HPT) axis by directly regulating TRH gene expression and, subsequently, TSH and thyroid hormone levels (18). Obesity-related increases in leptin levels can disrupt the HPT axis and affect thyroid hormone production resulting in an increase in the production of TSH (81, 82). Under normal conditions, increased leptin levels should prevent hyperphagia by stimulating anorexigenic peptides and suppressing orexigenic peptides in the hypothalamus (95). However, in obesity, a downregulation of leptin’s signaling pathways is observed (4, 95). Dysregulation of energy balance and metabolism results in leptin resistance or tolerance (96). Leptin resistance can occur either as a defect in leptin transportation across the blood-brain barrier or through intracellular inhibitory mechanisms (95, 97). Leptin resistance is a hallmark of diet-induced obesity (95). This is evidenced by numerous studies using human and rodent models (97, 98). Peripheral leptin resistance due to defective serum leptin transportation across the blood-brain barrier is associated with diet-induced obesity (99, 100). Peripheral leptin resistance occurs after initiating high-fat feeding and at a time when rodents are still responsive to centrally located leptin (99, 100). Presumably, peripheral resistance could result from elevated triglycerides interference with leptin blood-brain barrier transport, resulting in insufficient leptin levels within the brain (95, 100, 101). Furthermore, several studies have shown that chronically elevated leptin levels are pro-inflammatory, thus increasing the production of cytokines such as Tnf –α and IL– 6 (26). Interestingly, the HFHC diet-fed animals demonstrated a significant increase in leptin and IL-6 concentrations in conjunction with increased caloric intake and body weight compared to the control group, suggesting that the HFHC group demonstrates mild leptin resistance and impaired satiety. This could be due to the dual role IL-6 potentially plays in counteracting leptin resistance in the hypothalamus. These results are in agreement with the trends seen in obesity studies, where prolonged high-fat feeding led to impaired leptin signaling and hyperphagia (24, 102, 103).

It is important to note that the relationship between obesity and thyroid hormones is complex, and not all individuals with obesity will experience significant changes in thyroid function (76, 88). Moreover, the extent and nature of these changes can vary among obese individuals. Insulin-resistant obesity, also known as MUO, has been shown in the literature to cause major to severe dysregulation of the thyroid homeostatic pathways over time. Insulin resistance has further been demonstrated to be a catalyst in exacerbating the severity of homeostatic dysregulation, resulting in organ damage and dysregulated metabolic homeostasis. In contrast, MHO, which includes a state of preserved insulin sensitivity, is beneficial in counteracting many of the detrimental effects associated with the development of obesity. As evidenced by results from this study, we can speculate that due to the preserved state of insulin sensitivity, the detrimental metabolic and hormonal effects commonly associated with MUO were nullified to a certain extent in this study due to the compensatory mechanisms carried out by hormones leptin and insulin and cytokine IL-6.

This study did have some limitations. Due to time and funding constraints, we could not perform a histopathological analysis of the thyroid gland, which would have given us a morphological understanding of the effects of MHO on thyroid function. Further investigation into the oxidative status of the thyroid gland and the receptor expression of insulin, leptin and IL-6 would have been beneficial in providing an in-depth understanding of the mechanisms that aid in preventing severe thyroid homeostatic dysregulation in the metabolically healthy obese state. However, the collective results obtained in this study warrant using this diet-induced obese animal model to study further the phenotypical and genotypical changes that occur during the metabolically healthy obese state. The methodological procedures carried out in this study and the subsequent results obtained have validated the reliability and reproducibility of this particular combination diet-induced obese animal model to emulate the natural history and the metabolic characteristics of the human state of metabolically healthy obesity.

## 5 Conclusion

In conclusion, the effects associated with HFHC diet-induced obesity on thyroid homeostasis include elevated plasma TSH, fT_3_ and insulin concentrations. This was accompanied by elevated BMI, fasting blood glucose, HOMA-IR index, glycated hemoglobin, plasma IL-6, leptin and fT_4_ levels. However, the elevated concentrations of fasting blood glucose, HOMA-IR index, glycated hemoglobin, and insulin reported in the HFHC diet group compared to the control diet group did not fully satisfy the minimum criteria to be characterized as a metabolically unhealthy obesity subtype as mentioned above. These observations suggest that thyroid homeostasis is disturbed in the obese state. However, thyroid hormones and anabolic hormone insulin compensate to a certain extent for the changes to leptin and glucose signaling to maintain a degree of insulin sensitivity. Furthermore, altered levels of thyroid function markers in the obese state may lead to the development of insulin resistance initially and not thyroid disorders. The glucose homeostatic parameters assessed in the HFHC diet group further emphasize insulin’s monumental role in maintaining overall metabolic homeostasis. Conclusively, our study demonstrates that a dietary combination of high fats and high carbohydrates could effectively develop an animal model that mimics the natural history and metabolic characteristics of metabolically healthy obesity in humans. Therefore, we suggest that this model can be used to understand further the homeostatic changes that occur during the metabolically healthy obese state which can open new avenues for developing preventative methods for avoiding the progression to metabolically unhealthy obesity.

## Data Availability

The original contributions presented in the study are included in the article/**Supplementary Material**. Further inquiries can be directed to the corresponding author.

## References

[1] Ruiz S Vázquez F Pellitero S Puig-Domingo M. Endocrine obesity: Pituitary dysfunction in obesity. Eur J Endocrinology. (2022) 186:R79–92. doi: 10.1530/EJE-21-0899 35333754

[2] Lobstein T Brinsden H Neveux M. World obesity atlas 2022. (2022).

[3] Gong J Shen Y Zhang H Cao M Guo M He J. Gut microbiota characteristics of people with obesity by meta-analysis of existing datasets. Nutrients. (2022) 14:2993. doi: 10.3390/nu14142993 35889949 PMC9325184

[4] Rabiu AM Wale H Garba K Sabo AM Hassan Z Shugaba AI. Body mass index of male and female Wistar rats following administration of leptin hormone after a dietary regime. Ann Bioanthropology. (2017) 5(1):22. doi: 10.4103/aoba.aoba_17_16

[5] Bansal N. Prediabetes diagnosis and treatment: A review. World J diabetes. (2015) 6:296–303. doi: 10.4239/wjd.v6.i2.296 25789110 PMC4360422

[6] Laville M Nazare J-A. Diabetes, insulin resistance and sugars. Obes Rev. (2009) 10:24–33. doi: 10.1111/j.1467-789X.2008.00562.x 19207533

[7] Bessesen DH. The role of carbohydrates in insulin resistance. J Nutr. (2001) 131:2782S–6S. doi: 10.1093/jn/131.10.2782S 11584106

[8] Srivastava AK Pandey SK. Potential mechanism(s) involved in the regulation of glycogen synthesis by insulin. Mol Cell Biochem. (1998) 182:135–41. doi: 10.1023/A:1006857527588 9609122

[9] Murdolo G Bartolini D Tortoioli C Piroddi M Iuliano L Galli F. Lipokines and oxysterols: novel adipose-derived lipid hormones linking adipose dysfunction and insulin resistance. Free Radical Biol Med. (2013) 65:811–20. doi: 10.1016/j.freeradbiomed.2013.08.007 23954331

[10] Shimano H. Novel qualitative aspects of tissue fatty acids related to metabolic regulation: lessons from Elovl6 knockout. Prog Lipid Res. (2012) 51:267–71. doi: 10.1016/j.plipres.2011.12.004 22266797

[11] Frühbeck G Toplak H Woodward E Yumuk V Maislos M Oppert JM. Obesity: the gateway to ill health - an EASO position statement on a rising public health, clinical and scientific challenge in Europe. Obes facts. (2013) 6:117–20. doi: 10.1159/000350627 PMC5644725 23548858

[12] Novelli ELB Diniz YS Galhardi CM Ebaid GMX Rodrigues HG Mani F. Anthropometrical parameters and markers of obesity in rats. Lab Animals. (2007) 41:111–9. doi: 10.1258/002367707779399518 17234057

[13] Wilding JPH. Endocrine testing in obesity. Eur J Endocrinol. (2020) 182:C13–c5. doi: 10.1530/EJE-20-0099 32061161

[14] Reinehr T. Obesity and thyroid function. Mol Cell endocrinology. (2010) 316:165–71. doi: 10.1016/j.mce.2009.06.005 19540303

[15] García-Solís P García OP Hernández-Puga G Sánchez-Tusie AA Sáenz-Luna CE Hernández-Montiel HL. Thyroid hormones and obesity: a known but poorly understood relationship. Endokrynologia Polska. (2018) 69:292–303. doi: 10.5603/EP.2018.0032 29952420

[16] Cicatiello AG Di Girolamo D Dentice M. Metabolic effects of the intracellular regulation of thyroid hormone: old players, new concepts. Front Endocrinol (Lausanne). (2018) 9:474. doi: 10.3389/fendo.2018.00474 30254607 PMC6141630

[17] Nilsson M Fagman H. Development of the thyroid gland. Development. (2017) 144:2123–40. doi: 10.1242/dev.145615 28634271

[18] Kapoor P Kumar P Kapoor A. Thyroid, obesity and thyromimetic compounds. Indian J Clin Pract. (2018) 29:622–31.

[19] Kyriacou A Tziaferi V Toumba M. Stress, thyroid dysregulation, and thyroid cancer in children and adolescents: proposed impending mechanisms. Hormone Res Paediatrics. (2023) 96:44–53. doi: 10.1159/000524477 35385843

[20] Biondi B. Thyroid and obesity: an intriguing relationship. J Clin Endocrinol Metab. (2010) 95(8):3614–7. 10.1210/jc.2010-1245 20685890

[21] Volke L Krause K. Effect of thyroid hormones on adipose tissue flexibility. Eur Thyroid J. (2021) 10:1–9. doi: 10.1159/000508483 PMC7983599 33777816

[22] Walczak K Sieminska L. Obesity and thyroid axis. Int J Environ Res Public Health. (2021) 18:9434. doi: 10.3390/ijerph18189434 34574358 PMC8467528

[23] Ding X Zhu C-Y Li R Wu L-P Wang Y Hu S-Q. Lower normal free thyroxine is associated with a higher risk of metabolic syndrome: a retrospective cohort on Chinese population. BMC Endocrine Disord. (2021) 21:39. doi: 10.1186/s12902-021-00703-y PMC7934401 33663458

[24] Dornbush S Aeddula NR. Physiology, Leptin. In: StatPearls. StatPearls Publishing, Treasure Island (FL). (2025). 30725723

[25] Kelesidis T Kelesidis I Chou S Mantzoros CS. Narrative review: the role of leptin in human physiology: emerging clinical applications. Ann Intern Med. (2010) 152:93–100. doi: 10.7326/0003-4819-152-2-201001190-00008 20083828 PMC2829242

[26] Pérez-Pérez A Toro A Vilariño-García T Maymó J Guadix P Dueñas JL. Leptin action in normal and pathological pregnancies. J Cell Mol Med. (2018) 22:716–27. doi: 10.1111/jcmm.2018.22.issue-2 PMC5783877 29160594

[27] Spira D Buchmann N Dörr M Markus MRP Nauck M Schipf S. Association of thyroid function with insulin resistance: data from two population-based studies. Eur Thyroid J. (2022) 11:e210063. doi: 10.1530/ETJ-21-0063 35085102 PMC8963174

[28] Chooi YC Ding C Magkos F. The epidemiology of obesity. Metabolism: Clin experimental. (2019) 92:6–10. doi: 10.1016/j.metabol.2018.09.005 30253139

[29] Martinez B Ortiz RM. Thyroid hormone regulation and insulin resistance: insights from animals naturally adapted to fasting. Physiology. (2017) 32:141–51. doi: 10.1152/physiol.00018.2016 28202624

[30] Biondi B Kahaly GJ Robertson RP. Thyroid dysfunction and diabetes mellitus: two closely associated disorders. Endocr Rev. (2019) 40:789–824. doi: 10.1210/er.2018-00163 30649221 PMC6507635

[31] Iacobini C Pugliese G Blasetti Fantauzzi C Federici M Menini S. Metabolically healthy versus metabolically unhealthy obesity. Metabolism: Clin experimental. (2019) 92:51–60. doi: 10.1016/j.metabol.2018.11.009 30458177

[32] Cho YK Lee YL Jung CH. Pathogenesis, murine models, and clinical implications of metabolically healthy obesity. Int J Mol Sci. (2022) 23:9614. doi: 10.3390/ijms23179614 36077011 PMC9455655

[33] Blüher M. Metabolically healthy obesity. Endocrine Rev. (2020) 41(3):bnaa004. doi: 10.1210/endrev/bnaa004 32128581 PMC7098708

[34] Achilike I Hazuda HP Fowler SP Aung K Lorenzo C. Predicting the development of the metabolically healthy obese phenotype. Int J Obes (2005). (2015) 39:228–34. doi: 10.1038/ijo.2014.113 PMC4351862 24984752

[35] King AJ. The use of animal models in diabetes research. Br J Pharmacol. (2012) 166:877–94. doi: 10.1111/j.1476-5381.2012.01911.x PMC3417415 22352879

[36] Lee JS Jun DW Kim EK Jeon HJ Nam HH Saeed WK. Histologic and metabolic derangement in high-fat, high-fructose, and combination diet animal models. Sci World J. (2015) 2015(1):306326. doi: 10.1155/tswj.v2015.1 PMC4451512 26090514

[37] Kanasaki K Koya D. Biology of obesity: lessons from animal models of obesity. J Biomedicine Biotechnol. (2011) 2011:197636. doi: 10.1155/bmri.v2011.1 PMC3022217 21274264

[38] Hariri N Thibault L. High-fat diet-induced obesity in animal models. Nutr Res Rev. (2010) 23:270–99. doi: 10.1017/S0954422410000168 20977819

[39] Faine LA Diniz YS Almeida JA Novelli EL Ribas BO. Toxicity of ad lib. overfeeding: effects on cardiac tissue. Food Chem toxicology: an Int J published Br Ind Biol Res Assoc. (2002) 40:663–8. doi: 10.1016/S0278-6915(02)00004-2 11955672

[40] Lozano I van der Werf R Bietiger W Seyfritz E Peronet C Pinget M. High-fructose and high-fat diet-induced disorders in rats: impact on diabetes risk, hepatic and vascular complications. Nutr Metab (Lond). (2016) 13:15. doi: 10.1186/s12986-016-0074-1 26918024 PMC4766713

[41] Wong SK Chin KY Suhaimi FH Fairus A Ima-Nirwana S. Animal models of metabolic syndrome: a review. Nutr Metab (Lond). (2016) 13:65. doi: 10.1186/s12986-016-0123-9 27708685 PMC5050917

[42] Luvuno M Mabandla M Khathi A. Voluntary ingestion of a high-fat high-carbohydrate diet: a model for prediabetes. Ponte Int Sci Res J. (2018) 74. doi: 10.21506/j.ponte.2018.5.11

[43] Neto JGO Romão JS. Fructose consumption induces molecular adaptations involving thyroid function and thyroid-related genes in brown adipose tissue in rats. Braz J Med Biol Res. (2023) 55:e12240. doi: 10.1590/1414-431x2022e12240 36651452 PMC9843734

[44] Shao SS Zhao YF Song YF Xu C Yang JM Xuan SM. Dietary high-fat lard intake induces thyroid dysfunction and abnormal morphology in rats. Acta pharmacologica Sinica. (2014) 35:1411–20. doi: 10.1038/aps.2014.82 PMC4220075 25263336

[45] El-Sayed SM Ibrahim HM. Effect of high-fat diet-induced obesity on thyroid gland structure in female rats and the possible ameliorating effect of metformin therapy. Folia Morphologica. (2020) 79:476–88. doi: 10.5603/FM.a2019.0100 31489607

[46] Gaur A Pal GK Pal P. Role of ventromedial hypothalamus in sucrose-induced obesity on metabolic parameters. Ann Neurosci. (2021) 28:39–46. doi: 10.1177/09727531211005738 34733053 PMC8558980

[47] Swarnalatha NB Roy N Gouda MM Moger R Abraham A. High-fat, simple-carbohydrate diet intake induces hypothalamic–pituitary–thyroid axis dysregulation in C57BL/6J male mice. Appl Physiology Nutrition Metab. (2018) 43:371–80. doi: 10.1139/apnm-2017-0410 29099999

[48] Clemente-Suárez VJ Beltrán-Velasco AI Redondo-Flórez L Martín-Rodríguez A Tornero-Aguilera JF. Global impacts of western diet and its effects on metabolism and health: A narrative review. Nutrients. (2023) 15:2749. doi: 10.3390/nu15122749 37375654 PMC10302286

[49] Parasuraman S Raveendran R Kesavan R. Blood sample collection in small laboratory animals. J Pharmacol pharmacotherapeutics. (2010) 1:87–93. doi: 10.4103/0976-500X.72350 PMC3043327 21350616

[50] Geloneze B Vasques A Stabe C Pareja J Rosado L Queiroz ED. HOMA1-IR and HOMA2-IR indexes in identifying insulin resistance and metabolic syndrome - Brazilian Metabolic Syndrome Study (BRAMS). Arquivos brasileiros endocrinologia e metabologia. (2009) 53:281–7. doi: 10.1590/S0004-27302009000200020 19466221

[51] Levy JC Matthews DR Hermans MP. Correct homeostasis model assessment (HOMA) evaluation uses the computer program. Diabetes Care. (1998) 21:2191–2. doi: 10.2337/diacare.21.12.2191 9839117

[52] Mabuza LP Gamede MW Maikoo S Booysen IN Ngubane PS Khathi A. Effects of a ruthenium schiff base complex on glucose homeostasis in diet-induced pre-diabetic rats. Molecules. (2018) 23(7):1721. doi: 10.3390/molecules23071721 30011905 PMC6100054

[53] Mabuza LP Gamede MW Maikoo S Booysen IN Ngubane PS Khathi A. Cardioprotective effects of a ruthenium (II) Schiff base complex in diet-induced prediabetic rats. Diabetes Metab Syndr Obes. (2019) 12:217–23. doi: 10.2147/DMSO.S183811 PMC6385740 30858714

[54] Gamede M Mabuza L Ngubane P Khathi A. The effects of plant-derived oleanolic acid on selected parameters of glucose homeostasis in a diet-induced pre-diabetic rat model. Molecules (Basel Switzerland). (2018) 23:794. doi: 10.3390/molecules23040794 29596390 PMC6017303

[55] Singla P Bardoloi A Parkash AA. Metabolic effects of obesity: A review. World J diabetes. (2010) 1:76–88. doi: 10.4239/wjd.v1.i3.76 21537431 PMC3083889

[56] Cordain L Eaton SB Sebastian A Mann N Lindeberg S Watkins BA. Origins and evolution of the Western diet: health implications for the 21st century. Am J Clin Nutr. (2005) 81:341–54. doi: 10.1093/ajcn.81.2.341 15699220

[57] Kojta I Chacińska M Błachnio-Zabielska A. Obesity, bioactive lipids, and adipose tissue inflammation in insulin resistance. Nutrients. (2020) 12(5):1305. doi: 10.3390/nu12051305 32375231 PMC7284998

[58] Parmar RM Can AS. Physiology, appetite and weight regulation. In StatPearls [Internet]. Treasure Island (FL): StatPearls Publishing. (2025). 34662053

[59] Barrett KE Barman SM Brooks HL Yuan JXJ. Endocrine Functions of the Pancreas & Regulation of Carbohydrate Metabolism. Ganong's Review of Medical Physiology. 26e. New York, NY: McGraw-Hill Education (2019).

[60] Wang G. Raison d'être of insulin resistance: the adjustable threshold hypothesis. J R Society Interface. (2014) 11:20140892. doi: 10.1098/rsif.2014.0892 PMC4223910 25320065

[61] Tsatsoulis A Paschou SA. Metabolically healthy obesity: criteria, epidemiology, controversies, and consequences. Curr Obes Rep. (2020) 9:109–20. doi: 10.1007/s13679-020-00375-0 32301039

[62] Swinburn BA Sacks G Hall KD McPherson K Finegood DT Moodie ML. The global obesity pandemic: shaped by global drivers and local environments. Lancet. (2011) 378:804–14. doi: 10.1016/S0140-6736(11)60813-1 21872749

[63] Sears B Perry M. The role of fatty acids in insulin resistance. Lipids Health disease. (2015) 14:121–. doi: 10.1186/s12944-015-0123-1 PMC4587882 26415887

[64] Johnson JD. On the causal relationships between hyperinsulinaemia, insulin resistance, obesity and dysglycaemia in type 2 diabetes. Diabetologia. (2021) 64:2138–46. doi: 10.1007/s00125-021-05505-4 34296322

[65] Guemes M Rahman SA Hussain K. What is a normal blood glucose? Arch Dis childhood. (2016) 101:569–74. doi: 10.1136/archdischild-2015-308336 26369574

[66] Nkosi A Pather R Mshengu B Khathi A Ngubane P. Establishing a female animal model of prediabetes using a high-carbohydrate, high-fat diet. Curr Issues Mol Biol. (2024) 46:12397–416. doi: 10.3390/cimb46110736 PMC11592985 39590330

[67] Vyakaranam S Vanaparthy S Nori S Palarapu S Bhongir AV. Study of insulin resistance in subclinical hypothyroidism. Int J Health Sci Res. (2014) 4(9):147–53. PMC4286301 25580384

[68] Yang W Jin C Wang H Lai Y Li J Shan Z. Subclinical hypothyroidism increases insulin resistance in normoglycemic people. Front Endocrinol. (2023) 14. doi: 10.3389/fendo.2023.1106968 PMC10358968 37484968

[69] Ngema M Xulu ND Ngubane PS Khathi A. Pregestational prediabetes induces maternal hypothalamic–pituitary–adrenal (HPA) axis dysregulation and results in adverse foetal outcomes. Int J Mol Sci. (2024) 25:5431. doi: 10.3390/ijms25105431 38791468 PMC11122116

[70] Koga M. 1, 5-Anhydroglucitol and glycated albumin in glycemia. Adv Clin Chem. (2014) 64:269–301. 24938022 10.1016/b978-0-12-800263-6.00007-0

[71] Care D. Care in diabetes—2022. Diabetes Care. (2022) 45:S17. doi: 10.2337/dc22-S002 34964875

[72] Tura A Grespan E Göbl CS Koivula RW Franks PW Pearson ER. Profiles of glucose metabolism in different prediabetes phenotypes, classified by fasting glycemia, 2-hour OGTT, glycated hemoglobin, and 1-hour OGTT: an IMI DIRECT study. Diabetes. (2021) 70:2092–106. doi: 10.2337/db21-0227 34233929

[73] Boursier G Sultan A Molinari N Maimoun L Boegner C Picandet M. Triglycerides and glycated hemoglobin for screening insulin resistance in obese patients. Clin Biochem. (2018) 53:8–12. doi: 10.1016/j.clinbiochem.2017.12.002 29225096

[74] Zhang N Wang G. From metabolically healthy obesity to metabolically unhealthy obesity populations: decreased bone turnover bioactivity. Diabetes Metab Syndrome Obes. (2023) 16:3657–67. doi: 10.2147/DMSO.S431194 PMC10659145 38028998

[75] Zhang X Chen W Shao S Xu G Song Y Xu C. A high-fat diet rich in saturated and mono-unsaturated fatty acids induces disturbance of thyroid lipid profile and hypothyroxinemia in male rats. Mol Nutr Food Res. (2018) 62:1700599. doi: 10.1002/mnfr.201700599 29363248

[76] Laurberg P Knudsen N Andersen S Carlé A Pedersen I Karmisholt J. Thyroid function and obesity. Eur Thyroid J. (2012) 1:159–67. doi: 10.1159/000342994 PMC3821486 24783015

[77] Sosa-López J Alarcón-González P Sánchez-Hernández V Cruz-Estrada A Aguilar-Serralde C Velasco-Medina A. Impact of obesity on the thyroid profile, long-term experience at the General Hospital of Mexico, Ó?Dr. Eduardo LiceagaÓ. Rev Med del Hosp Gen Mexico. (2021) 84(1):4–10. doi: 10.24875/HGMX.20000012

[78] Longhi S Radetti G. Thyroid function and obesity. J Clin Res Pediatr Endocrinol. (2013) 5(Suppl 1):40. doi: 10.4274/jcrpe.856 PMC3608008 23149391

[79] Mele C Mai S Cena T Pagano L Scacchi M Biondi B. The pattern of TSH and fT4 levels across different BMI ranges in a large cohort of euthyroid patients with obesity. Front Endocrinol. (2022) 13. doi: 10.3389/fendo.2022.1029376 PMC9606412 36313780

[80] Marzullo P Minocci A Mele C Fessehatsion R Tagliaferri M Pagano L. The relationship between resting energy expenditure and thyroid hormones in response to short-term weight loss in severe obesity. PloS One. (2018) 13:e0205293. doi: 10.1371/journal.pone.0205293 30339686 PMC6195261

[81] Kurylowicz A Jonas M Lisik W Jonas M Wicik ZA Wierzbicki Z. Obesity is associated with a decrease in expression but not with the hypermethylation of thermogenesis-related genes in adipose tissues. J Transl Med. (2015) 13:31. doi: 10.1186/s12967-015-0395-2 25622596 PMC4314800

[82] Fontenelle LC Feitosa MM Severo JS Freitas TECD Morais JBS Torres-Leal FL. Thyroid function in human obesity: underlying mechanisms. Hormone Metab Res = Hormon- und Stoffwechselforschung = Hormones metabolisme. (2016) 48 12:787–94. doi: 10.1055/s-0042-121421 27923249

[83] Amouzegar A Kazemian E Gharibzadeh S Mehran L Tohidi M Azizi F. Association between thyroid hormones, thyroid antibodies and insulin resistance in euthyroid individuals: A population-based cohort. Diabetes Metab. (2015) 41:480–8. doi: 10.1016/j.diabet.2015.04.004 26049821

[84] Kocatürk E Kar E Küskü Kiraz Z Alataş Ö. Insulin resistance and pancreatic β cell dysfunction are associated with thyroid hormone functions: A cross-sectional hospital-based study in Turkey. Diabetes Metab Syndrome: Clin Res Rev. (2020) 14:2147–51. doi: 10.1016/j.dsx.2020.11.008 33395774

[85] Hu X Liu Y Wang C Hou L Zheng X Xu Y. Metformin affects thyroid function in male rats. Oncotarget. (2017) 8:107589–95. doi: 10.18632/oncotarget.22536 PMC5746091 29296189

[86] Abiri B Ahmadi AR Mahdavi M Amouzegar A Valizadeh M. Association between thyroid function and obesity phenotypes in healthy euthyroid individuals: an investigation based on Tehran Thyroid Study. Eur J Med Res. (2023) 28:179. doi: 10.1186/s40001-023-01135-1 37248529 PMC10228016

[87] Ambrosi B Masserini B Iorio L Delnevo A Malavazos A Morricone L. Relationship of thyroid function with body mass index and insulin-resistance in euthyroid obese subjects. J endocrinological Invest. (2010) 33:640–3. doi: 10.1007/BF03346663 20339314

[88] Alina K. Endocrine Disorders Accompanying Obesity - Effect or Cause? In: Venketeshwer R Leticia R, editors. Role of Obesity in Human Health and Disease. IntechOpen, Rijeka (2021). p. 2.

[89] Lang X Li Y Zhang D Zhang Y Wu N Zhang Y. FT3/FT4 ratio is correlated with all-cause mortality, cardiovascular mortality, and cardiovascular disease risk: NHANES 2007-2012. Front Endocrinology. (2022) 13:964822. doi: 10.3389/fendo.2022.964822 PMC9433660 36060933

[90] Lang X Zhao B Fang S Li L Li Z Wu N. Higher peripheral thyroid sensitivity is linked to a lower risk of heart failure after acute myocardial infarction. J Clin Endocrinol Metab. (2023) 108:2950–60. doi: 10.1210/clinem/dgad240 PMC10584000 37104944

[91] Skurk T Alberti-Huber C Herder C Hauner H. Relationship between adipocyte size and adipokine expression and secretion. J Clin Endocrinol Metab. (2007) 92:1023–33. doi: 10.1210/jc.2006-1055 17164304

[92] Schuett H Luchtefeld M Grothusen C Grote K Schieffer B. How much is too much? Interleukin-6 and its signalling in atherosclerosis. Thromb haemostasis. (2009) 102:215–22. doi: 10.1160/TH09-05-0297 19652871

[93] Bobbo VC Engel DF Jara CP Mendes NF Haddad-Tovolli R Prado TP. Interleukin-6 actions in the hypothalamus protects against obesity and is involved in the regulation of neurogenesis. J Neuroinflammation. (2021) 18:192. doi: 10.1186/s12974-021-02242-8 34465367 PMC8408946

[94] Freitas Lima LC Braga VDA do Socorro de França Silva M Cruz JDC Sousa Santos SH de Oliveira Monteiro MM. Adipokines, diabetes and atherosclerosis: an inflammatory association. Front Physiol. (2015) 6:304–. doi: 10.3389/fphys.2015.00304 PMC4630286 26578976

[95] Scarpace PJ Zhang Y. Leptin resistance: a prediposing factor for diet-induced obesity. Am J Physiol Regulatory Integr Comp Physiol. (2009) 296:R493–500. doi: 10.1152/ajpregu.90669.2008 PMC2665845 19091915

[96] Myers MG Cowley MA Münzberg H. Mechanisms of leptin action and leptin resistance. Annu Rev Physiol. (2008) 70:537–56. doi: 10.1146/annurev.physiol.70.113006.100707 17937601

[97] Yang R Barouch LA. Leptin signaling and obesity. Circ Res. (2007) 101:545–59. doi: 10.1161/CIRCRESAHA.107.156596 17872473

[98] Levin BE Dunn-Meynell AA. Reduced central leptin sensitivity in rats with diet-induced obesity. Am J Physiol Regulatory Integr Comp Physiol. (2002) 283:R941–8. doi: 10.1152/ajpregu.00245.2002 12228064

[99] Prpic V Watson PM Frampton IC Sabol MA Jezek GE Gettys TW. Differential mechanisms and development of leptin resistance in A/J versus C57BL/6J mice during diet-induced obesity. Endocrinology. (2003) 144:1155–63. doi: 10.1210/en.2002-220835 12639896

[100] Banks W Clever C Farrell CL. Impaired transport of leptin across the blood-brain barrier in obesity is acquired and reversible. Am J Physiol Endocrinol Metab. (2003) 285:E10–E5. doi: 10.1152/ajpendo.00468.2002 12618361

[101] Widdowson PS Upton R Buckingham R Arch J Williams G. Inhibition of food response to intracerebroventricular injection of leptin is attenuated in rats with diet-induced obesity. Diabetes. (1997) 46:1782–5. doi: 10.2337/diabetes.46.11.1782 9356026

[102] Genchi VA D'Oria R Palma G Caccioppoli C Cignarelli A. Impaired leptin signalling in obesity: is leptin a new thermolipokine? Int J Mol Sci. (2021) 22(12):6445. doi: 10.3390/ijms22126445 34208585 PMC8235268

[103] Girard J. Is leptin the link between obesity and insulin resistance? Diabetes Metab. (1997) 23 Suppl 3:16–24. 9342538

