# Supplementary material for: The effects of obesity on thyroid function in a metabolically healthy high-fat, high-carbohydrate diet-induced obese rat model
Source: Front Endocrinol (Lausanne). 2025 Apr 22;16:1538627. doi: 10.3389/fendo.2025.1538627 (PMC12052566; doi:10.3389/fendo.2025.1538627)
Supplement: Supplementary file 1 [file Table1.docx]

**Supplementary Table 1:** Baseline assessment

|  | **Diet group** | |
| --- | --- | --- |
|  | **Control diet** | **HFHC diet** |
| **(n) animals** | 9 | 9 |
| **Body weight (g)** | 183±1.75 | 189±1.22 |
| **Fasting blood glucose (mmol/L)** | 4.2±0.59 | 4.2±0.77 |
| **OGTT**  **2-hr post-prandial (mmol/L)** | 4.4±0.36 | 4.5±0.81 |
| **Cholesterol (mmol/L)** | 2.3±0.08 | 2.6±0.05 |
| **Triglyceride (mmol/L)** | 1.0±0.31 | 1.1±0.25 |
| **Food calorie intake (Kcal/g)** | 81±0.67 | 80±0.51 |
| **Water calorie intake (Kcal/ml)** | 0 | 0 |
| **Total calorie intake (Kcal/g)** | 81±0.67 | 80±0.51 |

Values are expressed as mean ± SEM (n=9 in each group).
